# Distribution of Prizes at St. Thomas's Hospital Medical School

**Published:** 1904-07-02

**Authors:** 


### DISTRIBUTION OF PRIZES AT ST. THOMAS'S HOSPITAL MEDICAL SCHOOL.

On Friday, 24th ult., in the presence of a large and distinguished assembly, the prizes, medals, and certificates for the year were presented to the successful students by Sir Thomas Barlow, K.C.V.O., D.Sc., LL.D.

The Treasurer, Mr. J. G. Wainwright, in opening the proceedings, drew attention to the structural improvements recently completed and to those now in progress, including the Nurses' Home, specially mentionening the lately renovated wards Albert, George, and Arthur, which, he believed, were now as perfect in every detail as could be seen anywhere. The expenditure was large, but would have to be increased still further owing to the need for increased and improved out-patient accommodation, particularly in relation to the special departments for diseases of the ear, throat, and skin, with regard to which he hoped that, before the next prize-giving, a well-matured scheme would be placed before the Governors. During the year 72,000 individuals received treatment, making 174,799 attendances.

The prizes were then distributed by Sir Thomas Barlow, followed by the presentation of medals, the medalists being introduced by the Dean of the Medical School. Mr. K. Takaki was the winner of the Mead Medal for Practical Medicine and of the Treasurer's Gold Medal for General Proficiency and Good Conduct; Mr. A. G. Gibson took the Wainwright and the Seymour Graves Toller Prize for Practical Medicine; Mr. H. A. Kisch the Cheselden Medal for Surgery and Surgical Anatomy; Mr. A. E. Boycott the Grainger Testimonial Prize for Anatomical and Physiological Research; and Mr. H. R. Dean the Solly Medal for Reports of Surgical Cases. Certificates of Proficiency and Certificates of Honour were also presented for the year 1903–1904.

### SIR THOMAS BARLOW'S ADDRESS.

Sir Thomas Barlow congratulated the prize-winners on their success, remarking that, despite criticism, prize-giving day must always be a red-letter day to each successive generation of students, so long as they were made of good human stuff and retained a store of generous enthusiasm. To the unsuccessful he would say, Do not be discouraged because of failure in one branch, since it may be an indication of success in another. He urged successful and unsuccessful alike not to lay aside examinations, but to use the store of material which preparation had necessitated for future elaboration in their own way, since even a humble and unpublished contribution to scientific knowledge might be helpful in the study of the natural history of some common disease and in the practical relief of some human suffering. By having some line of inquiry always ready for fresh reading, observation, and experiment, not only was special knowledge gained, but receptivity for fruitful knowledge in general was educated. *Viva voce* examinations were especially valuable, not only for the opportunities they afforded of linking what he might call brute facts together, and of profiting to the full by failure before others, but because the capacity for terse and forcible speech, lucid explanation, and facility in the art of persuasion (the true test of oratory, as Cobden said), and, not least, the cure of self-consciousness were among their fruitful effects. Speaking to those about to enter upon professional life outside the hospital, Sir Thomas Barlow counselled avoidance of the cynical view that a young doctor must aim primarily to please his patients and to avoid all appearance of doubt or hesitancy, especially at the expense of his professional brethren. Though worldly wisdom and *savoir faire* were not to be despised, there was a more excellent way for the young doctor, and such qualities might well be left to the period of disillusionment that came with middle life. Keen enthusiasm for knowledge gained in the laboratory and wards and the enjoyment of taking a humble part in the successful results of surgical skill, or in following the guidance of rational medicine, the learning to know, as the basis of intelligent action—this was the spirit to be cherished. Diagnosis was nine-tenths of private as of hospital practice, and when from the nature of the case that was incomplete, the wise man would avoid heroic remedies and keep to simple therapeutics. In the ardour for investigation it must not, however, be forgotten that the smallest details for the relief of pain were worthy of thought and study, and that clothing, food, and exercise admitted of scientific regulation, as well as the quantity and combinations in medicine. Of collateral methods of learning which were of enormous value in practice he would first refer to the systematic and exhaustive study and record of blunders, whether inevitable, depending upon present limitations of knowledge; or avoidable, consequent on taking things for granted instead of reviewing and revising from day to day. Even the loneliness experienced in early active private practice had its compensations in the wholesome if unenlightened criticisms from which illumination sometimes came. Scientific intercourse should be maintained, and the habit of terse and exhaustive note-taking, formed in hospital, cultivated. In these days of faith-healing, Christian science and hypnotism, and of charlatanism of every kind, inside the profession as well as outside, there were many pitfalls into which the young practitioner might tumble, and among them was the exaltation of his own personality and undue influence over those whom he had to treat. Although genuine sympathy, the determination to understand the outlook of others, and a broad and enlightened charity were in no other profession more appropriate, yet he would remind them that there was in hospital practice a certain brevity, a businesslike straightforwardness, and a simplicity in dealing with people which were very wholesome, and which appeared to be justified in their results. He thought the nearer that was approached to in private practice the better. Although subject to criticism there was a considerable area in which they were independent, and that independence should be jealously guarded. In the professional intercourse with people the plain, straightforward duty of finding out what was physically wrong and the effort to set it right must always be kept in view. That was the primary function of the doctor, and if it were kept primary the other parts of conduct might be trusted to find their proper place in the pattern of life. The perfect openness and outspokenness of the hospital rule was one of its perennial charms—no secret remedies, no confidences, everything above board, right out in the open. In the words of Bishop Ken—

> Let all thy converse be sincere,
> Thy conscience as the noonday clear.

After a vote of thanks to Sir Thomas Barlow, moved by

Dr. S. J. Sharkey and seconded by Mr. A. P. Boyson, the Treasurer, accompanied by members of the Grand Committee and by the Staff, escorted Sir Thomas Barlow through the hospital, the whole of which was open for inspection. Tea was served in the garden to a large number of visitors, and the band of his Majesty's Irish Guards gave selections of music at intervals.

